# Lead tolerance and siderophore production by native *Pseudomonas* spp. isolated from lead-contaminated environments in Colombia

**DOI:** 10.1007/s12223-025-01307-y

**Published:** 2025-08-13

**Authors:** Susana Ochoa-Agudelo, Jessica M. Bedoya-Vélez, Andrés F. Villa-Restrepo, J. Felipe Osorio-Tobón

**Affiliations:** 1 Faculty of Health Sciences, Department of Health Sciences, Biosciences Group, University Institution Colegio Mayor de Antioquia (IUCMA), Carrera 78 # 65 - 46 Bloque Patrimonial, Antioquia, Medellín, Colombia; 2 School of Microbiology, Biotransformation Group, University of Antioquia, Cl. 67 # 53–108 Bloque 5, Antioquia, Medellín, Colombia

**Keywords:** *Pseudomonas*, Lead, Siderophores, Pyoverdine production

## Abstract

Siderophores are organic compounds known for their chelating properties and specific biochemical characteristics, with potential applications in biotechnology, especially in bioremediation. This study evaluated lead tolerance and pyoverdine-type siderophore production in native *Pseudomonas* spp. isolates from lead-contaminated water. Lead tolerance was assessed using different lead concentrations (5, 10, 25, 50 mg/L) through the minimum inhibitory concentration. Siderophore production was estimated based on the percentage of siderophore units (PSU) in the presence and absence of lead. Among the 14 strains analyzed, 11 operational taxonomic units (OTUs) were identified. Four *Pseudomonas* spp. strains (P07, P35, P36, and P38) were tolerant to lead concentrations higher than 25 mg/L. Although pyoverdine production was detected in P07, P10, P11, P16, P25, P35, and P38 strains, no synthesis of catecholate or hydroxamate was detected. The P07 strain was the highest siderophore producer with a PSU of 90 ± 8.5%. Lead concentration above 600 ppm reduced the PSU by up to 34%. This study demonstrates that native strains of *Pseudomonas* isolated from lead-contaminated extreme environments can tolerate lead while producing bioactive metabolites, such as pyoverdine-type siderophores.

## Introduction

Lead is one of the five most toxic heavy metals that cause environmental problems. Different anthropogenic activities, such as pesticide use in agriculture and industrial activities associated with paint production and children’s toys, release lead into the environment (Atitsogbey et al. 2023). Pesticides produce different harmful effects on living organisms, including damage to the central nervous system, impairment of pulmonary function with gradual damage, hematological changes, liver damage, and cardiovascular dysfunction (Balali-Mood et al. 2021). For lead at the environmental level, the World Health Organization (WHO) establishes maximum permissible limits between 0.10 and 0.17 mg/kg in the USA, Canada, Colombia, and other countries (Health Organization 2023). Moreover, the WHO has implemented a campaign that involves reducing the use of lead since 2020, promoting replacement, and controlling the amount of lead in environmental waste (World Health Organization 2022). In Colombia, concentrations of up to 23 mg/L found in rivers are higher than the limit established by the regulations (0.01–0.20 mg/L) (Resolution 0883 of 2018). Additionally, in Colombia, the amount of lead in materials such as paints and toys must not exceed 90 mg/L (Congreso de Colombia 2020).

Environmental control of heavy metals has focused on technologies that act on the principle of physicochemical properties such as membrane filtration and ion exchange (Shan et al. 2023). However, while these technologies are effective, they are not sustainable over time and become costly and unfeasible when applied on a large scale (Verma and Kuila 2019). For this reason, many researchers have identified an opportunity in biotechnological applications, seeking alternatives in the study of bacteria, fungi, and algae that coexist in environments where these heavy metals are abundant and could represent a solution for the toxic environment. For their development, these organisms of biological origin have developed mechanisms that allow them to survive in extreme environments containing heavy metals (Fakhar et al. 2022; Pal et al. 2022; Firincă et al. 2024).

Some research reports the use of microbial mechanisms as external characteristics associated with the cell membrane or the production of compounds that enable them to survive in polluted environments. Additionally, other mechanisms that depend on the metabolic capacity of each microorganism can be associated (Atitsogbey et al. 2023). This study focuses on the ability of bacteria of the genus *Pseudomonas* spp. to grow in metal-polluted environments. Some studies have explored the ability of *Pseudomonas* to tolerate the presence of heavy metals, including different mechanisms like biosorption, bioprecipitation, or bioaccumulation (Beni and Esmaeili 2020; Tang et al. 2024; Olaya-Abril et al. 2024). The genus *Pseudomonas* is an effective biosorbent due to its ability to produce exopolysaccharides (Kalita and Joshi 2017; Alabssawy and Hashem 2024), along with other metabolites such as lipoproteins and siderophores, which primarily chelate metals like iron (Saati-Santamaría et al. 2022).

Siderophores are low-molecular-weight peptidic molecules with a high affinity for iron. These molecules are vital in bacterial mechanisms related to survival in metal-deficient media (Soares 2022). Siderophores are produced by different organisms such as bacteria, fungi, plants, and others. They are classified into chemical groups according to the iron chelating functional group. For example, they are classified as catecholates, hydroxymates, phenolates, carboxylate, or mixed group siderophores, which contain more than one functional group, such as pyoverdine synthesized by *Pseudomonas aeruginosa* (Swayambhu et al. 2021; Roskova et al. 2022). There are more than 500 types of siderophores reported, and their differences are related to the affinity for iron, oxidative capacity, stability with metal determined by the K constant, and the stability in the formed siderophore-metal complex (Roskova et al. 2022). Some functional activities are focused on its virulent mechanism and are used as a competitive factor between pathogens of the specificity in the affinity for iron. Microbial siderophore producers are excellent competitors in removing iron from the host or any other pathogen that requires it (Qin et al. 2022). Additionally, in natural environments, the presence of siderophore-producing microorganisms supports the growth of microorganisms that contribute to plant development (Ghosh et al. 2020).

In *Pseudomonas* genera, siderophores have been extensively studied, among which pyoverdine and pyochelin are the main siderophores. Generally, both compounds are synthesized in very low concentrations of iron or iron absence (Ghssein and Ezzeddine 2022). However, the production of these compounds is also associated with the heavy metals presence and their interaction with them. Understanding the behavior of microorganisms in the presence of heavy metals can be a starting point for developing novel bioremediation strategies through the potential use of metabolites or the whole microorganism. The objective of this study was to evaluate the ability of microbial native isolates from wastewater compatible with the genus *Pseudomonas* to synthesize pyoverdine-type siderophores in the presence and absence of lead.

## Materials and methods

### Isolation and microbial identification

Wastewater samples were collected from an industrial area (6°23′02.9″ N 75°27′09.9″ W, Girardota, Antioquia, Colombia) (Framework Permit, Resolution 1467 of December 3, 2014). In the sample area, a lead concentration of 0.5 mg/L was previously detected (Ministerio de Ambiente y Desarrollo sostenible 2015). Initially, four isolates of the genus *Pseudomonas*, obtained in previous studies (Vélez et al. 2021), were characterized and identified by a unique strain code (P07, P10, P16, and P25). Then, to expand the number of isolates compatible with the *Pseudomonas* genus, ten additional isolates were obtained and designated by a unique strain code to identify each strain during the analysis (P11, P26, P27, P29, P30, P32, P34, P35, P36, and P38). Then, the isolates were characterized through morphological and biochemical tests using the VITEK2 Compact® identification System (bioMérieux, France). The isolates were subjected to phylogenetic analysis according to the methodology described by Vélez et al. (2021). The total DNA was extracted from the isolates using the ADN PowerSoil DNA Isolation Kit (MO BIO Laboratories, Inc., EE. UU.). The 16S rDNA gene sequence was amplified with primers 27 F (5′-AGAGAGTTTGATCCTGGCTCAG-3′) and 1492R (5′-GGTTACCTTGTTACGACTT-3′) using the amplification conditions described by Janssen (2006). PCR products were analyzed and purified using Wizard® PCR Preps (Promega, New England) and an automated sequencer ABI PRISM 3700 DNA (Applied Biosystems). Sequences were edited with the Software MEGA 10 and subjected to BLASTN (NCBI—National Center for Biotechnology Information (NCBI)) (Antil et al. 2023). Similarity analysis was performed using sequences from GenBank with percentages higher than 97%. The sequences generated were deposited in GenBank (NCBI). Additionally, isolates were subjected to GMYC speciation analysis to establish the clades according to operational taxonomic units (OTUS) (Luo et al. 2018). The isolates were conserved in a BHI medium (Merck®, USA) with 30% glycerol as a cryoprotective agent at − 20 °C until further analysis, all strains were deposited in the biologycal collection, CEMBIO from University Institution Colegio Mayor de Antioquia (IUCMA).

### Lead tolerance assay

To assess the tolerance of the isolates to the presence of lead, an exposure test of the microorganisms to different concentrations of lead nitrate solution—Pb (NO_3_)_2_—was performed; 200 µL of BHI medium (Merck®, USA) at concentrations of 0.5, 2.5, 5, 25, and 50 mg/L of the metal solution was evaluated in 96-well polystyrene plates. The isolates were incubated for 24 h at 32 ± 2 °C. A fresh culture was used as inoculum to obtain suspensions of each isolate with a cell density adjusted to a 0.5 McFarland standard (1 × 10^8^ cells/mL). Plates containing different concentrations of lead nitrate solution were inoculated with 20 µL of the suspensions of each isolate, obtaining a final volume in each well of 200 µL. BHI medium supplemented with the metal solution was used as a negative control. Moreover, *P. aeruginosa* ATCC 27853 was inoculated in BHI medium without metal solution and used as microbial control growth. Each assay was performed in triplicate. The minimum inhibitory concentration (MIC) was defined as the concentration of the lead solution at which a decrease in cell density was detected. Microorganisms with MIC values higher than that of the reference strain were considered lead-tolerant (Liu et al. 2023).

### Qualitative detection and chemical classification of siderophores

To identify siderophore production, a culture on chrome azurol S agar (CAS agar) was made for each strain and incubated at 32 °C for 24 h (Patel et al. 2018). To identify whether lead induces siderophore production, the isolates previously cultured in BHI medium were cultured in CAS agar supplemented with Pb (NO_3_)_2_ (0.5 mg/L). A yellow-to-orange halo around the colonies means a positive result for siderophore production (Neilands 1987).

Simultaneously, qualitative tests were conducted to identify the type of siderophores produced: hydroxamates, catecholates, or mixed ligands (e.g., pyoverdine type). Culture media lead supplemented and non-supplemented were incubated at 32 °C and 150 rpm for 24 h. The cultures were centrifuged at 10,000 rpm for 10 min. The supernatant was used to identify the chemical nature of the siderophore (Dell’Anno et al. 2022).

In the spectrophotometric method reported by Neilands (1987), 3 mL of a 2% aqueous FeCl_3_ solution was added to 1 mL of supernatant to detect the presence of hydroxamate-type siderophores. A peak between 420 and 450 nm indicates the presence of hydroxamate-type siderophores. The production of catecholates was determined using the method described by Arnow (1937). In this method, a red–orange solution indicates a positive result for this type of siderophore.

The detection of pyoverdine-type siderophores was performed on King B agar (Sigma—Aldrich®) and King B agar supplemented with Pb (NO_3_)_2_ (0.5 mg/L). Samples were incubated at 32 ± 2 °C for 48 h (Johnsen and Nielsen 1999). The synthesis of pyoverdine-type compounds is associated with flourishing greenish-yellow pigments when exposed to ultraviolet light (365 nm).

All assays were performed in triplicate. Uninoculated media were used as negative controls for the methods (Neilands 1987), while *P. aeruginosa* ATCC 27853 and *E. coli* ATCC 25922 were used as positive and negative reference strains, respectively.

### Siderophore activity

The siderophore activity was quantitatively estimated using the CAS reagent (Neilands 1987). Strains were incubated in MMS pyoverdine induction medium for 24 h. Then, 1 mL of supernatant was mixed with 1 mL of CAS reagent and incubated for 20 min (Vindeirinho et al. 2021). Absorbance at 630 nm was recorded to quantify siderophore activity (Thermo ScientificTM Genesys 180 UV–Vis). Reference samples were prepared as mentioned before, using 1 mL of uninoculated medium (Murakami et al. 2021). All assays were conducted in triplicate, with uninoculated media serving as negative controls. The quantitative activity of the siderophore produced for each strain was expressed as a percentage of siderophore units (PSU) according to Eq. (1):

1 $${\mathrm{PSU}}=\frac{Ar-As}{Ar}\times 100$$

where *Ar* and *As *represent the reference and sample absorbance, respectively.

#### Pyoverdine-type siderophore production in the presence of lead

Isolates were selected according to the tolerance lead assay and siderophore activity. The selected strains were cultivated in BHI medium and MMS medium with succinic acid as an inductor for pyoverdine-type siderophore production. Lead concentrations of 0, 100, 400, 600, 800, and 1000 ppm were used in BHI medium, whereas concentrations of 0, 10, 100, and 1000 ppm were used in MMS medium (Vindeirinho et al. 2021).

A suspension of each strain was prepared and adjusted to a turbidity equivalent to 0.5 on the McFarland standard scale; 100 mL of each medium was inoculated with 5% of each microbial suspension. Experimental units were incubated at 32 ± 2 °C at 150 rpm for 24 h. An aliquot from each sample was taken, and the absorbance was determined at an optical density of 600 nm to determine biomass. Subsequently, samples were centrifuged at 10,000 rpm for 10 min. Siderophore production was estimated in the supernatant by measuring absorbance at 405 nm, according to Beer’s Law (Vindeirinho et al. 2021) Siderophore capacity to chelate iron was determined by measuring the PSU (Murakami et al. 2021).

### Statistical analysis

A descriptive analysis was performed to assess lead tolerance. An ANOVA was conducted at a significance level of 0.05 to evaluate siderophore production. All analyses were carried out in triplicate, and the data were analyzed using Minitab® v.16 software.

## Results and discussion

### Characterization and identification of microbial isolates

**Fig. 1:**
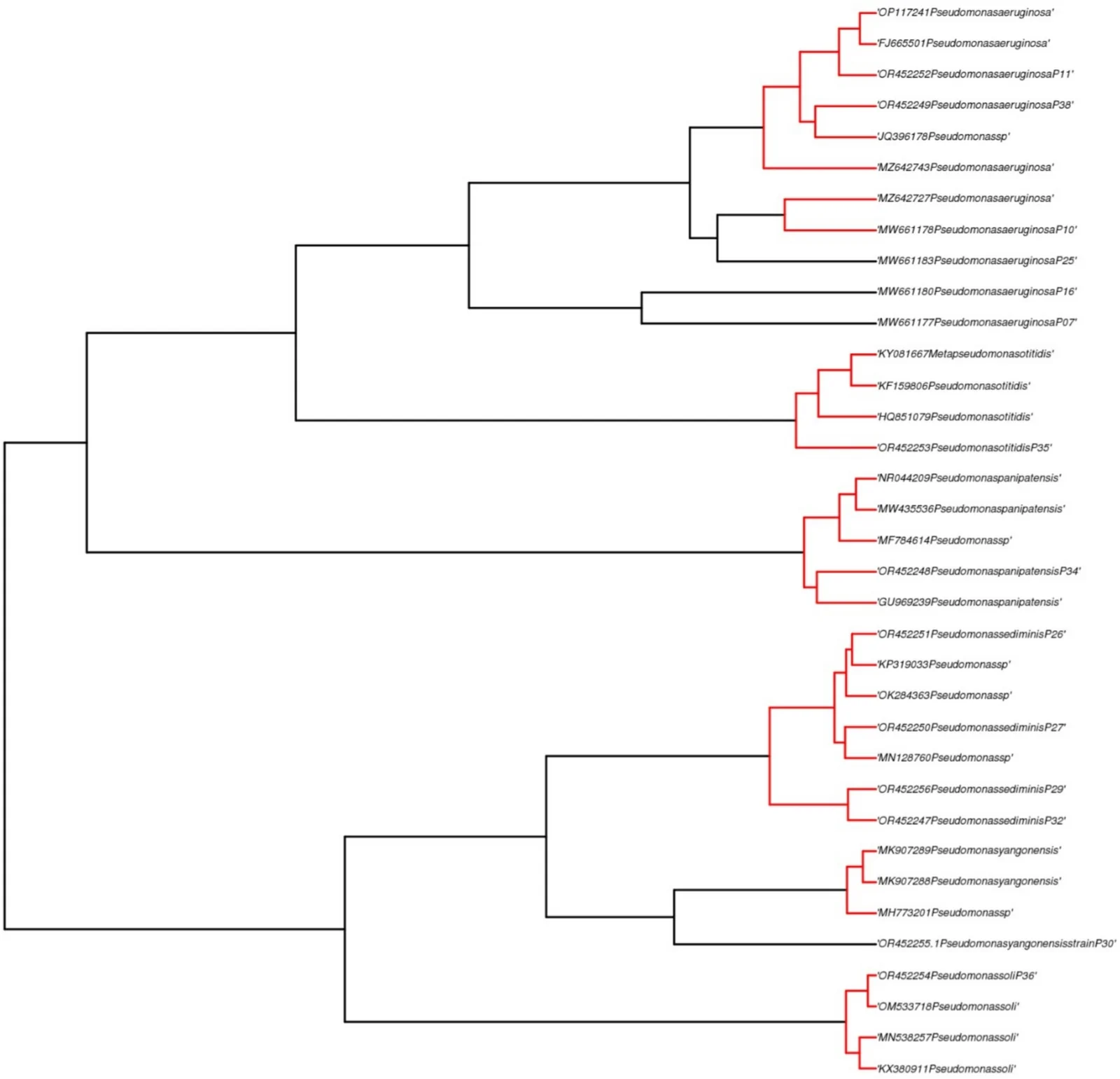
Phylogenetic analysis of native *Pseudomonas* spp. isolated from lead-contaminated wastewater. Red lines indicate the clades in which isolates were grouped

All microorganisms exhibited a morphology consistent with Gram-negative bacilli and tested positive for oxidase. According to semi-automated biochemical tests, all ten new isolates belong to the genus *Pseudomonas* spp. Two of the new strains were consistent with *P. aeruginosa* (P11 and P38) with a probability greater than 95% (see Table 1). Analysis of the partial sequence of the 16S rRNA gene and GMYC delimitation corroborated the classification of the isolates within the genus *Pseudomonas*. As can be observed in Fig. 1, *P. aeruginosa* (P07, P10, P11, P16, P25, and P38), *P. otitidis* (P35), *P. panipatensis* (P34), *P. sediminis* (corresponding to P26, P27, P29, and P32), *P. soli* (P36), and *P. yagonesis* (P30) were identified. Among the six isolates identified as *Pseudomonas aeruginosa*, P10 and P25 exhibited distinct phenotypic characteristics. The separation of clades into OTUs indicates that the strains found exhibit distinct characteristics. For example, P11 and P38 belong to another clade; meanwhile, P07 and P16 were clustered into another. Moreover, P30 was identified as *P. yagonensis* and grouped in a different clade for the principal OTU (see Fig. 1, uncolored lines). The nucleotide sequences generated for the new ten isolates identified in this study were deposited in GenBank (NCBI) with the following accession codes: OR452247–OR452256 (available at https://submit.ncbi.nlm.nih.gov/subs/?search=SUB13765753).

**Table 1:** Characterization of lead-tolerant bacterial isolates based on morphological, biochemical, and molecular identification

**Strain code**	**Microscopy characteristics**		**Oxidase**	**Catalase**	**Biochemical identification (VITEK®)**	**Probability (%)**	**Molecular identification**	MIC** Pb (NO_3_)_2_ (mg/L)
	Gram	Form						
P07*	Negative	Bacillus	Positive	Negative	*P. aeruginosa*	98	*P. aeruginosa*	25
P10*	Negative	Bacillus	Positive	Negative	*P. aeruginosa*	98	*P. aeruginosa*	5
**P11**	Negative	Bacillus	Positive	Negative	*P. aeruginosa*	98	*P. aeruginosa*	5
P16*	Negative	Bacillus	Positive	Negative	*P. aeruginosa*	98	*P. aeruginosa*	5
P25*	Negative	Bacillus	Positive	Negative	*P. aeruginosa*	98	*P. aeruginosa*	5
**P26**	Negative	Bacillus	Positive	Negative	*Pseudomonas *spp.	98	*P. sediminis*	5
**P27**	Negative	Bacillus	Positive	Negative	*Pseudomonas *spp.	98	*P. sediminis*	5
**P29**	Negative	Bacillus	Positive	Negative	Undefined	-	*P. sediminis*	5
**P30**	Negative	Bacillus	Positive	Negative	Undefined	-	*P. yagonensis*	5
**P32**	Negative	Bacillus	Positive	Negative	*Pseudomonas *spp.	98	*P. sediminis*	5
**P34**	Negative	Bacillus	Positive	Negative	*Pseudomonas *spp.	98	*P. panipatensis*	5
**P35**	Negative	Bacillus	Positive	Negative	Undefined	-	*P. otitidis*	25
**P36**	Negative	Bacillus	Positive	Negative	*Pseudomonas *spp.	50	*P. soli*	50
**P38**	Negative	Bacillus	Positive	Negative	*P. aeruginosa*	98	*P. aeruginosa*	25

*Isolates identified in previous study (Bedoya Vélez et al. 2021)

**MIC minimum inhibitory concentration, in lead presence, resulting in complete inhibition of isolate growth (Muñoz et al. 2012)

Regarding metal tolerance, different microbial mechanisms facilitate the interaction between microorganisms and metals, including enzymatic synthesis and the production of compounds such as proteins, peptides, lipids, and others associated with membrane-level flow pumps. *Pseudomonas* spp., *Bacillus* spp., Actinomycetes, and *Penicillium* sp. are some metal-tolerant microorganisms reported in some investigations (Tang et al. 2024). Among these microorganisms, *P. aeruginosa*, *P. fluorescens*, and *P. syringae* are some of the most recognized and studied species for their versatility in responding to metal tolerance, resistance, and metal removal in different ecosystems (Olaya-Abril et al. 2024; Alabssawy and Hashem 2024).

In this study, the finding of microorganisms corresponding to the genus *Pseudomonas* in lead-contaminated wastewater demonstrates the ability of the genus to adapt and survive in this type of environment. The detection of species such as *P. aeruginosa* is notable due to its capacity to remove metals (Shan et al. 2023).

### Lead tolerance

Table 1 presents the MIC of lead at which each microorganism remained viable. All isolates were tolerant to concentrations up to 5 mg/L of lead, whereas P07, P35, P36, and P38 showed higher lead tolerance than the other isolates. These strains showed lead tolerance concentrations between 25 and 50 mg/L, suggesting differential physiological or genetic adaptations to metal stress. These results are consistent with reports in the literature. For example, Al Disi and collaborators (2022) demonstrated the tolerance of *Pseudomonas* and *Bacillus* strains to heavy metal (Cr, Cu, Cd, and Pb) concentrations up to 50 mg/L. Moreover, Kelany and collaborators (2023) used *Enterobacter* spp. in monoculture or consortium to remove Zn^+2^, Fe^+2^, Pb^+2^, Co^+2^, Mn^+2^, Ni^+2^, and Cd^+2^, reporting a high percentage of metal removal, even at concentrations of 100 mg/L. Indigenous *Pseudomonas aeruginosa* and *Enterobacter aerogenes* strains remove heavy metals like Cu, Cd, and Pb from wastewater (Mazhar Shah 2025).

### Chemical classification and chelating capacity of siderophores

**Fig. 2:**
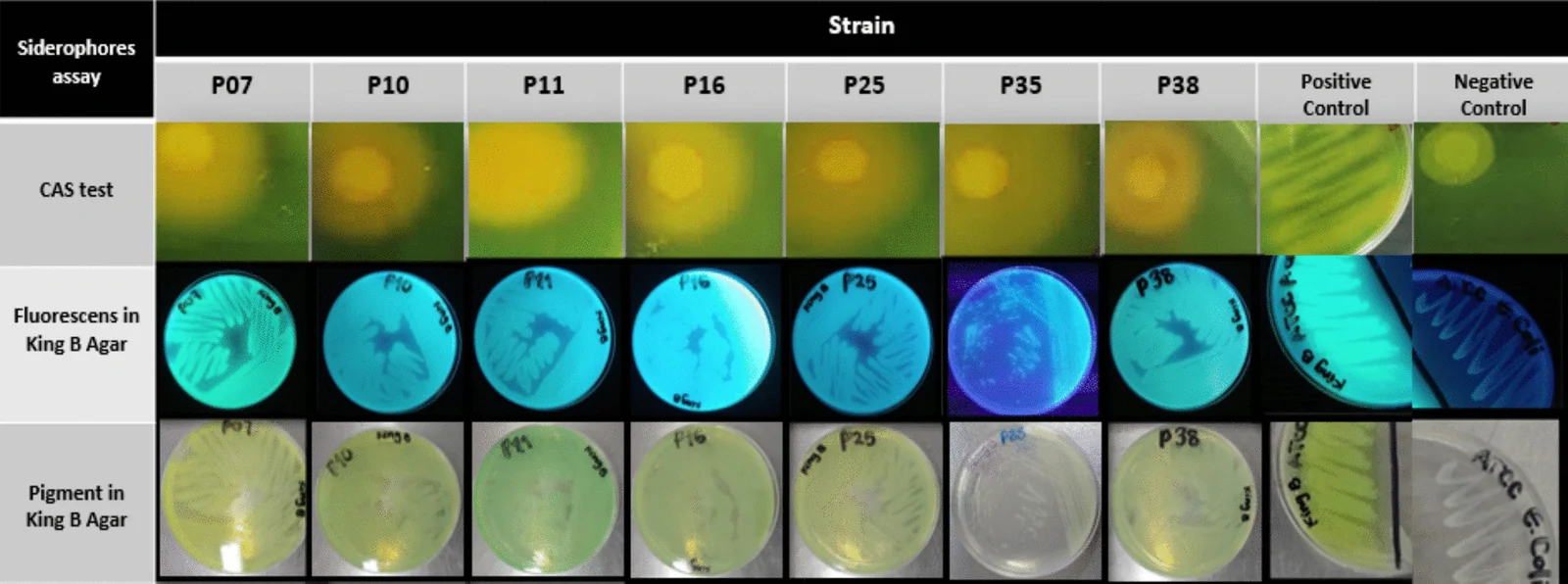
Siderophore production assessed by the CAS assay and colony characteristics of *Pseudomonas *isolates grown on King B agar

Figure 2 shows the results from CAS and King B agar for siderophore production. In CAS agar, pyoverdine production is indicated by a yellow halo around the colony; whereas in King B agar, pyoverdine production is detected by a yellow-greenish fluorescent color when exposed to UV rays (Microbienne et al. 2018). This confirmed the presence of siderophores produced by the genus *Pseudomonas* (Meyer 2000; Cézard et al. 2019). In this study, 50% of the isolates (P07, P10, P11, P16, P25, P35, and P38) exhibited siderophore-producing activity. Complementary to this assay, all isolates were previously incubated in a medium with lead solution (5 mg/L Pb (NO_3_)_2_) to stimulate siderophore production. Again, 50% of the isolates showed the production of a yellow halo around the colony.

In *Pseudomonas* spp., iron deficiency promotes the synthesis of pyoverdine-type siderophores due to their high affinity for iron (Murakami et al. 2021; Ghssein and Ezzeddine 2022). Among siderophores, metallophores are low molecular weight molecules that interact with different metals. Maldonado-Hernandez and collaborators (2022) demonstrated that metals like iron enhance the production of metallophores in CAS culture medium. The presence or absence of iron or another metal could be considered a regulatory factor for siderophore synthesis (Dell’Anno et al. 2022). In this work, although lead was added as an inductor, as shown in Table 2, no differences between lead-supplemented and non-lead-supplemented media were observed in the production of the siderophores in each test evaluated to classify the siderophores. Pyoverdine synthesis was detected in P07, P10, P11, P16, P25, P35, and P38 strains. On the contrary, no production of catecholate or hydroxamate was observed in any strain. The CAS culture medium does not identify the functional group of the siderophore, and additional analytical tests are required. In the same context, no absorbance peaks between 420 and 450 nm were detected for hydroxamates. Although hydroxamates and catecholates are siderophores with distinct ligand structures with better stability due to their cyclic configuration (Roskova et al. 2022; Soares 2022), no color change was observed for catecholate detection (see Table 2). According to the qualitative tests to classify the siderophores, the native isolates only produce a single siderophore type—likely pyoverdine independent of the lead stress. Therefore, the siderophore synthesis in these strains could be regulated primarily by iron availability rather than lead exposure.

**Table 2:** Chemical classification of siderophores produced by *Pseudomonas* spp

Chemical classification						
Strain code	Catecholate		Hydroxamate		Pyoverdine	
	Arnow test		Neyland’s test		King’s B	
	Lead (-)	Lead (+)	Lead (-)	Lead (+)	Lead (-)	Lead (+)
P07	-	-	-	-	+	+
P10	-	-	-	-	+	+
P11	-	-	-	-	+	+
P16	-	-	-	-	+	+
P25	-	-	-	-	+	+
P26	-	-	-	-	-	-
P27	-	-	-	-	-	-
P29	-	-	-	-	-	-
P30	-	-	-	-	-	-
P32	-	-	-	-	-	-
P34	-	-	-	-	-	-
P35	-	-	-	-	+	+
P36	-	-	-	-	-	-
P38	-	-	-	-	+	+

As can be observed in Table 3, all isolates grew in the King B agar test. However, fluorescence production was observed in strains P07, P10, P11, P16, P25, P35, and P38 (Fig. 2), while P26, P27, P29, P30, P32, P34, and P36 strains did not show fluorescence. A complementary screening test was performed to stimulate the production of siderophores. A King B agar supplemented with lead (5 mg/L Pb (NO_3_)_2_) was used. P07, P10, P11, P16, P25, P35, and P38 strains, which are siderophore producers on CAS culture medium, showed phenotypic characteristics, pigment, and fluorescence related to pyoverdine production in the presence of lead. The King B agar assay confirmed the synthesis of mixed-type siderophores, allowing the classification of *Pseudomonas* isolates into fluorescent and non-fluorescent groups.

**Table 3:** Screening and quantitative analysis of siderophore production and pigment expression

Strain code	Screnning CAS agar					
	CAS agar	Supplemented with lead	Viability	Pigment production	Fluorescence production	PSU (%)
**P07**	(+)	(+)	(+)	(+)	(+)	90 ± 8.50^a^
**P10**	(+)	(+)	(+)	(+)	(+)	83 ± 10.60^ab^
**P11**	(+)	(+)	(+)	(+)	(+)	79 ± 15.60^ab^
**P16**	(+)	(+)	(+)	(+)	(+)	81 ± 7.00^ab^
**P25**	(+)	(+)	(+)	(+)	(+)	72 ± 22.00^bc^
**P26**	(-)	(-)	(+)	(-)	(-)	NA
**P27**	(-)	(-)	(+)	(-)	(-)	NA
**P29**	(-)	(-)	(+)	(-)	(-)	NA
**P30**	(-)	(-)	(+)	(-)	(-)	NA
**P32**	(-)	(-)	(+)	(-)	(-)	NA
**P34**	(-)	(-)	(+)	(-)	(-)	NA
**P35**	(+)	(+)	(+)	(+)	(+)	44 ± 1.00^d^
**P36**	(-)	(-)	(+)	(-)	(-)	NA
**P38**	(+)	(+)	(+)	(+)	(+)	58 ± 1.00^c^
***P. aeruginosa* ATCC 27853 (Positive control)**	(+)	(+)	(+)	(+)	(+)	NA
***E.coli* ATCC 25922 (Negative control)**	(-)	(-)	(-)	(-)	(-)	NA

***Means followed by different letters are significantly different (p ≤ 0.05)***

CAS assay measures iron chelation, and the chelating capacity can be associated with higher values of PSU determined by this assay. Moreover, higher values of PSU are associated with an increased ability of the microorganism to sequester iron. PSU values ranging from 44 to 90% were obtained, as shown in Table 3. Although strains P26, P27, P29, P30, P32, and P34 showed no siderophore activity, strains P07, P10, P11, P16, P25, P35, and P38 showed siderophore activity. These strains synthesize these metabolites in the presence of heavy metals other than iron, according to the microbial ability to synthesize metallophores related to the mechanisms of adaptation to the environment. These results are consistent with the findings of Maldonado-Hernandez and collaborators (2022) who reported metallophore production in *Pseudomonas* strains ranging from 32.29 to 94.08% PSU in media supplemented with lead concentrations up to 10 mM. On the other hand, as siderophore activity measured by the PSU is not directly proportional to the amount of siderophore produced by each strain, and the synthesis of the siderophores could change when metals different than iron are present in the medium (Murakami et al. 2021), we carried out further experiments using the isolates P07 and P38 to explore the effect of the lead on pyoverdine production. These isolates were selected based on their siderophore activity and growth capacity in the presence of lead.

### Effect of lead on pyoverdine production

Strains P07 and P38 were selected to evaluate the effect of the lead on biomass production and pyoverdine production. For this purpose, LB medium was used as an enrichment culture medium, and MMS supplemented with succinic acid served as an induction medium for siderophore production. As can be observed in Table 4, values of biomass between 0.57 ± 0.08 and 1.17 ± 0.17 were obtained. According to the Tukey test, differences among culture media were observed, where biomass obtained by MMS medium is significantly lower than the LB medium for almost all lead concentrations. In LB medium, the biomass decreases as lead concentration increases for both strains. Although LB medium is an enrichment medium and provides nutrients to enhance biomass production, the metal represents an inhibitory effect on biomass production, mainly in the P38 strain. On the other hand, the lead concentration showed no influence on the biomass of strains P07 and P38 when MMS medium was used. MMS is a low-enrichment medium used to stimulate the synthesis of siderophores when supplemented with succinic acid. Vindeirinho and collaborators (2021) compared the use of MMS and King B media, establishing that the carbon source and the amount of succinic acid play a significant role in metabolite synthesis. Additionally, the presence of phosphate sources in the medium contributed to the stabilization of the soluble siderophore. Moreover, in this work, biofilm production was detected in the MMS culture medium when the lead concentrations were increased. There is a relationship between the synthesis of siderophores and the production of exopolysaccharides as protective mechanisms under stress conditions (Díaz-Pérez et al. 2022). For example, Cornelis and collaborators (2022) used different mutations in the *pvd* gene clusters responsible for siderophore synthesis. The synthesis of these metabolites is involved in the structure of biofilm production and the synthesis of other compounds directly or indirectly involved in quorum sensing systems (Jeong et al. 2023). Moreover, the production of biofilms as protectors of microbial growth is related to the synthesis of phenazines, generation of ROS, and acquisition of metals such as iron (Hao et al. 2021).

**Table 4:** Effect of lead on biomass and pyoverdine production in strains P07 and P38

Culture medium	Strain code	Lead concentration (ppm)	Biomass (DO_600_ nm)	Pyoverdine (µg/L)	PSU
LB	P07	0	1.10 ± 0.08^a^	28.14 ± 5.24^a^	69.00 ± 2.94^a^
		400	1.10 ± 0.02^ab^	32.97 ± 1.15^a^	65.64 ± 4.06^a^
		600	0.90 ± 0.12^ab^	29.52 ± 3.08^a^	70.36 ± 2.63^a^
		800	0.84 ± 0.11^b^	32.04 ± 1.81^a^	43.75 ± 4.41^b^
		1000	1.17 ± 0.17^ab^	32.48 ± 1.82^a^	36.26 ± 0.55^b^
	P38	0	1.08 ± 0.05^a^	23.98 ± 2.38^a^	69.93 ± 1.96^ab^
		400	0.57 ± 0.08^b^	22.26 ± 4.43^a^	65.27 ± 3.18^b^
		600	0.78 ± 0.24^b^	22.73 ± 2.20^a^	71.35 ± 1.26^a^
		800	0.83 ± 0.10^b^	22.55 ± 1.63^a^	51.71 ± 2.38^c^
		1000	0.77 ± 0.08^b^	25.23 ± 1.44^a^	33.24 ± 1.37^d^
MMS	P07	0	0.49 ± 0.24^a^	39.18 ± 0.39^a^	79.79 ± 1.93^ab^
		10	0.38 ± 0.01^a^	29.41 ± 1.22^b^	77.18 ± 0.19^b^
		100	0.39 ± 0.04^a^	31.98 ± 3.33^b^	79.49 ± 4.79^b^
		1000	0.23 ± 0.05^a^	8.51 ± 0.71^c^	86.49 ± 0.69^a^
	P38	0	0.20 ± 0.09^a^	10.14 ± 1.88^a^	80.68 ± 1.45^a^
		10	0.18 ± 0.09^a^	8.61 ± 1.11^a^	68.68 ± 4.89^b^
		100	0.20 ± 0.07^a^	8.95 ± 0.22^a^	82.69 ± 0.78^a^
		1000	0.20 ± 0.05^a^	3.78 ± 1.45^b^	71.79 ± 1.84^b^

Means followed by different letters are significantly different (*p* ≤ 0.05)

The siderophore production ranged from 22.23 to 32.97 μg/L and 3.78 to 39.18 μg/L in LB and MMS medium, respectively. P07 strain showed the highest yield in MMS medium without exposure to the metal. No statistically significant differences (*p* > 0.05) in pyoverdine-type siderophore production were observed between metal concentrations and isolates grown in LB medium. However, when the MMS medium was used, siderophore production had a statistically significant difference (*p* < 0.05). In this work, the higher lead concentration, the lower the pyoverdine-type siderophore amount, mainly in P38. This result is similar to those found by Ghssein and Ezzeddine (2022) in siderophore synthesis in different microorganisms. In that work, while the quantity of metal in the medium increases, the siderophore production decreases, therefore its detection. On the other hand, Díaz-Perez and collaborators (2022) observed that the percentage of siderophore units (PSU) remains high as long as the siderophore present in the medium has a strong affinity for iron. Moreover, Maldonado-Hernandez and collaborators (2022) measured siderophores in the medium, with exposure to different heavy metals (Zn, Cd, Pb, Ni, among others), identifying that the siderophores present in the medium can interact with heavy metals other than iron, perceiving a low activity of siderophore units, because siderophores showed a lower affinity to iron when other metals were present in the supernatant.

As expected, the PSU is affected by the presence of lead, as presented in Table 4. Generally, at lower lead concentrations, the PSU did not change, but at lead concentrations higher than 600 ppm, the PSU decreased. This phenomenon is mainly observed in the P07 isolated cultured in LB medium. The PSU decreased from 70.36 ± 2.63% to 36.26 ± 0.55% when the lead concentration increased from 600 to 1000 ppm. Similar behavior was observed in the P38 strain cultured in the LB medium. The PSU decreased from 71.35 ± 1.26% to 33.24 ± 1.37%. Conversely, in the MMS medium, the lead concentration did not have a clear tendency in the influence of lead concentration on the PSU. In the MMS medium, the P38 strain showed a PSU of 71% at the maximum concentration of the metal. The lead addition may not enhance siderophore production because the regulatory pathways are primarily iron-responsive. Although the CAS reagent test contains iron and the siderophore reacts with it due to its high affinity, the lead could not efficiently be chelated by the siderophores. Therefore, it is possible that lead cannot act as a stimulus for siderophore synthesis. However, the results indicate that as lead concentration increases in the medium, the percentage of siderophore activity decreases. That suggests the possibility that the cell-free supernatant derived from *Pseudomonas* isolates is forming complexes with lead instead of iron, as discussed in the study published by Shan et al. (2023).

## Conclusions

*P. aeruginosa, P. otitidis, P. panipatensis, P. sediminis, P. soli*, and *P. yagonesis* were isolated from wastewater. These strains survive in toxic environments containing lead at concentrations ranging from 5 to 50 mg/L. Seven strains (P07, P10, P11, P16, P25, P35, and P38), which belonged to *Pseudomonas aeruginosa*, produced pyoverdine-type siderophores. The strains produced siderophores with characteristics similar to those reported for pyoverdines associated with colonies with yellow-greenish pigment and production of fluorescence in King B culture medium. The strains identified as *P. aeruginosa* exhibited PSU up to 90%. The strains demonstrated chelating capacity for iron, even if lead is present in the medium culture. Although an increase in lead concentration caused a decrease in biomass and siderophore production, the strains respond with protection and tolerance mechanisms, maintaining their siderophore activity. Even at higher lead concentrations, selective media supplemented with succinic acid served as an inducer for their production, especially the strain P38. *Pseudomonas* strains can produce siderophores that interact with lead in aqueous media, and they respond with different mechanisms, including biomass and pyoverdine production. Although this study confirmed the capability of *Pseudomonas* to tolerate lead and produce siderophores, further research is needed at the genetic level to elucidate the pathways involved in siderophore biosynthesis, their interaction with heavy metals, and the characterization of pyoverdine to enhance production. The ability of these microorganisms to survive in lead-polluted environments and synthesize secondary metabolites represents a potential platform for developing bioremediation strategies.

## Data Availability

We declare that the data presented were obtained from laboratory work and are available upon request.
